# Bayesian network analysis of cognitive impairment in empty-nest older adults: the role of living environment

**DOI:** 10.3389/fpubh.2025.1680988

**Published:** 2025-11-17

**Authors:** Shiji Zhang, Yanzhen Tian, Nina Feng, Jinxiu Li, Tao Zhang, Libang Deng, Jianjun Fu

**Affiliations:** 1 Zhuzhou Hospital Affiliated to Xiangya School of Medicine, Central South University, Zhuzhou, China; 2 Medical College of Jishou University, Jishou, China; 3 Wuhan Sixth Hospital, Wuhan, China

**Keywords:** living environment, empty-nest older adults, cognitive impairment, bayesian network, influencing factors

## Abstract

**Objective:**

Based on the Bayesian network, this study investigates the impact pathways of multidimensional factors related to the living environment—specifically housing factors, exposure to daily chemical agents, daily fuel use, air quality, and drinking water sources—on cognitive impairment in empty-nest older adults individuals. The aim is to identify key direct and indirect predictors and provide a foundation for targeted environmental interventions.

**Methods:**

The study utilized data from China’s 2018 Comprehensive Longitudinal Health Survey (CLHLS) to track health-affecting factors, including a sample of 5,961 empty-nest older adults individuals. Potential predictive variables were initially screened through univariate analysis, followed by further screening of significant variables using binary logistic regression. We constructed the Bayesian Network structure with R’s bnlearn package and made probability predictions using Netica.

**Results:**

The incidence of cognitive impairment among the empty-nest older adults individuals is 18.7%. Results from a binary logistic regression analysis suggest that several factors are associated with an increased risk of cognitive impairment in this population. These factors encompass living in rural areas, exposure to daily chemical agents, lack of access to piped natural gas, use of kerosene and coal, insufficient kitchen ventilation, presence of a musty smell in the living space, smoking, failure to open windows during winter, and consumption of untreated water. Furthermore, the results from a Bayesian network model indicate that smoking, the absence of piped natural gas, musty odors in the room, and exposure to daily chemical agents are directly related to cognitive impairment. In contrast, living in rural areas, drinking untreated water, using coal, not opening indoor windows during winter, inadequate kitchen ventilation, a lack of air purification devices, and reliance on kerosene are indirectly associated with cognitive impairment. Notably, older adults individuals at the highest risk of cognitive impairment (41.5%) are those who smoke, experience musty odors in their residences, are exposed to daily chemicals, and lack access to piped gas.

**Conclusion:**

Factors related to the living environment can influence the cognitive functions of empty-nest older adults individuals through multiple pathways. Therefore, strategies for preventing cognitive impairment should adopt a multifactorial and integrated approach, incorporating both community and home-based interventions.

## 1 Introduction

The global population is undergoing an unprecedented aging process, with China’s trend being particularly pronounced. According to data from China’s seventh national census, individuals aged 60 and above comprise 18.70% of the population. It is projected that by 2035, the older population will reach approximately 400 million, accounting for 30% of the total population (1, 2).

Empty-nest older adults individuals are defined as those aged 60 years and older who live alone or only with their spouse (3). As population aging intensifies, life expectancy increases, and family structures in China evolve due to urbanization, the number of empty-nest older adults individuals is rising annually. It is projected that by 2030, China will have as many as 200 million people in empty-nest households, with over 90% of older adult households falling into this category (4). The physical decline experienced by older adults individuals who have become empty-nesters is significantly greater than that of their non-empty-nest peers, and they face elevated levels of social isolation, a lack of emotional support, and insufficient health monitoring (5, 6). Research indicates that the incidence of cognitive impairment among empty-nest older adults individuals in China is as high as 15.5% (7). The deterioration of cognitive function in the older people not only exacerbates the progression of chronic diseases such as cardiovascular disease (8), diabetes (9), and malnutrition (10), which are caused by functional decline, but also creates a psychological-physiological vicious cycle through pathophysiological pathways mediated by psychosocial stress. This situation increasingly worsens the burden of care for the older population on society and families.

Research indicates that cognitive impairment among the older adults experiencing the empty-nest phenomenon is associated with various factors, including depression (11), social relationships (12), and living environment (13). Among these factors, the living environment stands out as a critical component, encompassing the safety, convenience, and age-appropriateness of the physical surroundings, as well as the supportiveness and interactivity of the community social environment (14). Mi et al. (15) discovered that prolonged exposure to hypoxia correlates with a gradual deterioration in cognitive function, with the extent of damage progressively increasing. Additionally, several studies have identified industrial emission sources, such as products of incomplete fuel combustion, composite exposure to atmospheric pollutants (PM2.5/PM10), and the accumulation of environmental toxins, including tobacco smoke, as significant environmental exposure factors contributing to neurodegenerative risks (16, 17). Conversely, individuals with impaired cognitive function significantly benefit from an environment conducive to well-being, which positively affects the proportion of active older adults (2, 18). However, the studies referenced primarily utilize linear regression models that focus on a single influencing factor to analyze its impact on cognitive function. While these studies illuminate the relationship between individual factors and cognitive function, they often fail to analyze the intricate interaction mechanisms among environmental, behavioral, and physiological variables. Cognitive function is typically influenced by a complex network of relationships among various risk factors, including the interconnections between these factors, which may produce an overall cumulative effect (19). Considering China’s unique urban–rural dual structure and its specific social and cultural context, there is a significant gap in the systematic and thorough exploration of how the independence and interaction of living environments affect the cognitive health of older adults in empty-nest situations.

Bayesian Networks (BN) effectively describe the interrelationships among various factors and serve as a modeling method for uncertainty reasoning based on probability theory. They illustrate the interdependencies among variables through directed acyclic graphs and quantify the strength of correlations using conditional probability tables (20). In investigating the impact of living environment factors on cognitive disorders in empty-nest older adults, BN can uncover the complex internal associations between risk factors and the pathways of influence among them, thereby providing methodological support for analyzing the dynamic relationship between the “living environment” and “cognitive outcomes.”

This study aims to analyze the mechanisms through which the living environment of empty-nest older adults influences cognitive impairment. It employs a Bayesian network model to examine the determinants of living environments, utilizing data from the 2018 Chinese Longitudinal Healthy Longevity Survey (CLHLS). By identifying key environmental risks and modifiable protective factors, this approach aims to inform community-based aging-friendly transformations, the design of smart smart older-adult care environments, and the development of social support networks. Ultimately, these efforts are expected to effectively delay or even prevent the onset and progression of cognitive impairment in empty-nest older adults, thereby reducing the substantial socio-economic costs associated with this condition. The findings will provide evidence-based insights for the formulation of age-friendly housing policies, ultimately offering new scientific intervention targets to address the challenges associated with aging.

## 2 Methods

### 2.1 Data source

The data for this study were derived from China’s 2018 Comprehensive Longitudinal Health Survey (CLHLS) database, organized by the Center for Healthy Aging and Development Research at Peking University. The survey covers 23 provinces in China and is nationally representative due to its random sampling methodology. Each participant provided informed consent prior to data collection. This study utilizes the most recent CLHLS data from 2018, which initially included 15,874 older adults aged 60 and above as participants.

**Figure 1:**
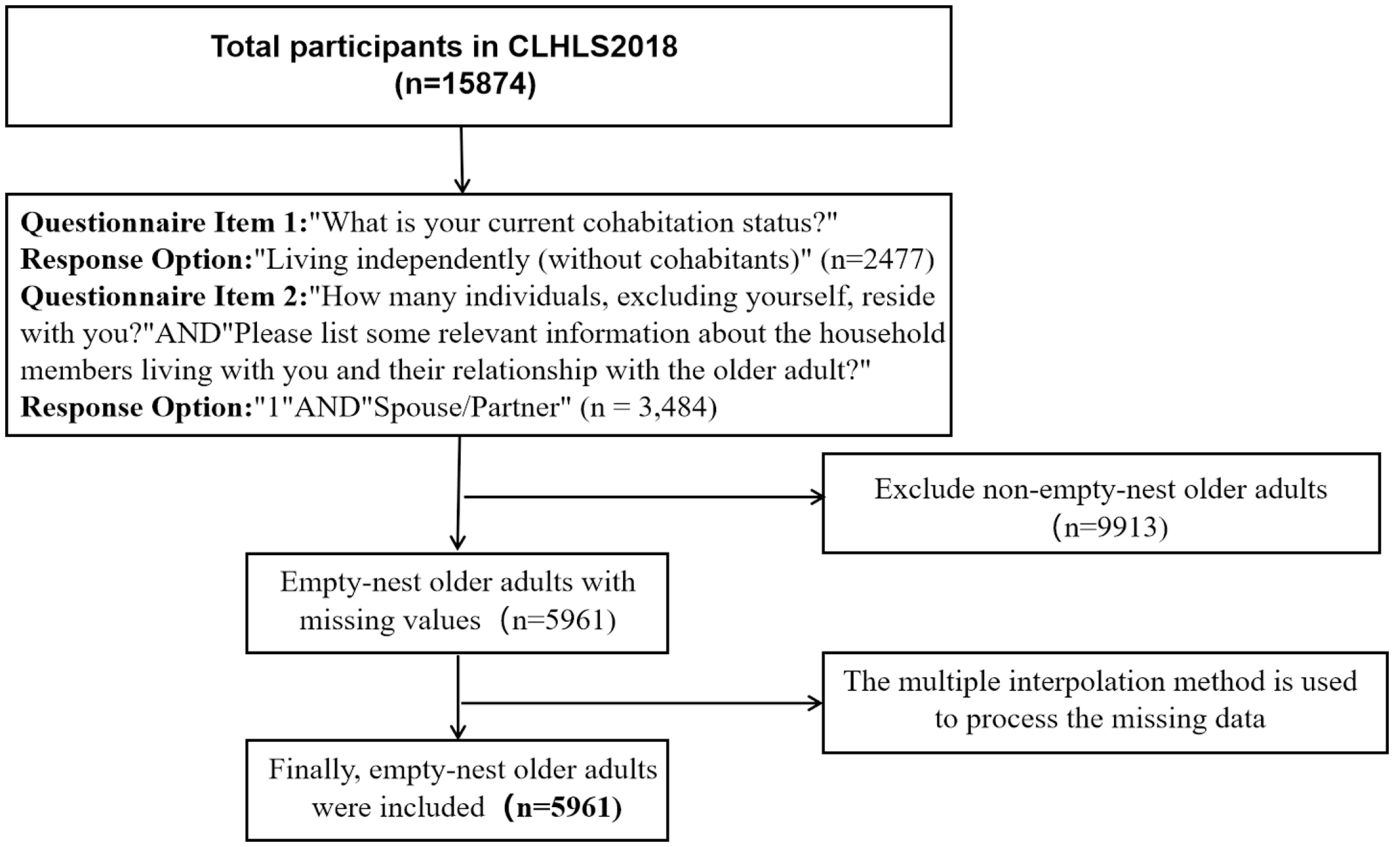
Research object selection process.

Based on the definition of empty-nest older adults, this study conducted the questionnaire entry “What is your current cohabitation status?” Choose “Living independently (without cohabitants)” and “How many individuals, excluding yourself, reside with you?” AND “Please list some relevant information about the household members living with you and their relationship with the older adult?” Choose “1” AND “Spouse/Partner” to determine the empty-nest older adults. The study focused on older adults aged 60 and above who met this definition as research subjects. To address missing data, a multiple imputation method was employed (21). The CLHLS study was approved by the Research Ethics Committee of Peking University (IRB00001052-13074), and ultimately included data from 5,961 older adults. For further details, refer to Figure 1.

### 2.2 Cognitive impairment assessment

This study employs cognitive impairment as the dependent variable. The Chinese Longitudinal Healthy Longevity Survey (CLHLS) assesses the cognitive function of older adults using the Chinese version of the Mini-Mental State Examination (MMSE), which encompasses five dimensions and a total of 24 items. These dimensions consist of general abilities (5 items), reaction abilities (3 items), attention and calculation abilities (6 items), recall abilities (3 items), and language comprehension and self-coordination abilities (6 items). The total score ranges from 0 to 30 points, with higher scores indicating better cognitive function. A score below 24 is classified as cognitive impairment, while a score of 24 or above is considered indicative of normal cognition (22).

### 2.3 Living environment assessment

This study, through a review of previous literature (2, 15–17, 21) and the CLHLS questionnaire, combined with group discussions, initially identified 21 potential related factors. These factors are categorized into five dimensions: housing, daily chemical exposure, fuel usage, air quality, and drinking water sources.

#### 2.3.1 Housing situation

This dimension includes six items: place of residence, housing type, the presence of a separate bedroom, the existence of roof leakage, distance from major traffic routes, and proximity to medical institutions. The Bayesian network model constructed in this study primarily addresses the relationships among categorical variables. To incorporate the continuous variables ‘distance from the main road’ and ‘distance from the medical institution’ into the model, we employed a discrete method to convert them into binary variables. Drawing on domain knowledge and previous research (16, 23), we categorized the distance from the main traffic road as ‘≤200 meters’ and ‘>200 meters’, while the distance from medical institutions was classified as ‘≤3 kilometers’ and ‘>3 kilometers’.

#### 2.3.2 Daily chemical exposure

This refers to whether there is contact with daily chemicals. The assessment of exposure to daily chemical agents is determined by the questionnaire item, “Do you use the following chemicals at home?” The response options are categorized as: None, Occasional, Sometimes, and Frequent. Responses indicating “None” and “Occasional” signify a lack of exposure to daily chemical agents, whereas responses of “Sometimes” and “Frequent” imply exposure. An individual is classified as exposed to daily chemical agents if they utilize any of the eight specified chemical agents, which include insecticides, moth repellents, air fresheners, air purifiers, disinfectants, toilet cleaning agents, and oil stain removal agents.

#### 2.3.3 Types of fuel used in daily life

This includes whether pipeline natural gas, kerosene, coal, or firewood is utilized.

#### 2.3.4 Indoor air quality

This encompasses kitchen ventilation, the use of air purification devices, the presence of musty odors in the room, smoking behavior, and whether windows are opened for ventilation across all four seasons: spring, summer, autumn, and winter. The decision to open windows for ventilation across these seasons was assessed through the questionnaire item: “In the past 12 months, how often have you opened the windows in your home for ventilation during spring, summer, autumn, and winter?” Responses indicating “Do not open the window” were categorized as “No,” while responses indicating “once or more” were categorized as “Yes.”

#### 2.3.5 Drinking water sources

This refers to the type and category of drinking water.

Table 1 provides detailed information on the assignment of all variables.

**Table 1:** Variable assignment table.

Dimensions	Variables		Assignment
Housing situation	Place of residence	X_1_	Rural = 0, City = 1
	Housing Type	X_2_	Bungalow = (0,0,0,0,0), Apartment on 1 ~ 3 floors = (0,1,0,0,0),≥4 floors apartment without elevator = (0,0,1,0,0),≥4 floors apartment with elevator = (0,0,0,1,0), Movable home = (0,0,0,0,0,1)
	Separate bedroom	X_3_	No = 0, Yes = 1
	Leaking rain	X_4_	No = 0, Yes = 1
	Distance from the main road	X_5_	≤200 meters = 0, > 200 meters = 1
	Distance from medical institutions	X_6_	≤3 kilometers = 0,>3 kilometers = 1
Daily chemical exposure	Exposure to daily chemical agents	X_7_	No = 0, Yes = 1
Types of fuel used in daily life	Use pipeline natural gas	X_8_	No = 0, Yes = 1
	Use kerosene	X_9_	No = 0, Yes = 1
	Use coal	X_10_	No = 0, Yes = 1
	Use firewood	X_11_	No = 0, Yes = 1
Indoor air quality	Kitchen ventilation	X_12_	No = 0, Yes = 1
	Use air purification device	X_13_	No = 0, Yes = 1
	moldy odor in the room	X_14_	No = 0, Yes = 1
	smoking behavior	X_15_	No = 0, Yes = 1
	Open windows for ventilation in spring	X_16_	No = 0, Yes = 1
	Open windows for ventilation in summer	X_17_	No = 0, Yes = 1
	Open windows for ventilation in autumn	X_18_	No = 0, Yes = 1
	Open windows for ventilation in winter	X_19_	No = 0, Yes = 1
Drinking water sources	Type of drinking water	X_20_	Well water = (0,0,0,0,0), River water or lake water = (0,1,0,0,0,), Spring water = (0,0,1,0,0), Pond water = (0,0,0,1,0), Tap water = (0,0,0,0,0,1)
	Category of drinking water	X_21_	Raw water = 0, Boiling water = 1

### 2.4 Covariates

Considering the impact of confounding factors, the covariates included in the analysis are gender (male or female), age (in years), living status (living alone or with others), marital status (with a spouse or unpartnered), and education level (illiterate or literate).

### 2.5 Statistical analysis

The collected data were summarized and entered into Excel software, with verification performed by two individuals. Statistical analysis was conducted using SPSS version 23.0, establishing a significance level of *p* < 0.05. In accordance with previous research, continuous numerical data were converted into categorical data to meet the requirements of the Bayesian network algorithm for discrete input, thereby simplifying the model structure and enhancing the interpretability of the results. Descriptive statistical analysis was conducted for general data. Potential predictor variables were identified through univariate analysis, and categorical data were assessed using the χ^2^ test, with the significance level set at *α* = 0.05 to identify statistically significant candidate variables. A binary logistic regression model was developed, followed by multifactor analysis, calculating the odds ratio (OR) for each variable along with its 95% confidence interval. Ultimately, based on the significant predictor variables identified through logistic regression, a Bayesian network model was constructed using the ‘bnlearn’ package in RStudio version 4.5.0. The max-min hill-climbing (MMHC) algorithm was employed for structure learning, while maximum likelihood estimation was utilized for parameter learning. Bayesian network inference was conducted using Netica version 5.18 software.

## 3 Results

### 3.1 General information on the distribution of empty-nest older adult population

This study included a total of 5,961 older adults from empty-nest households who met the inclusion criteria, consisting of 3,097 males (51.7%) and 2,882 females (48.3%). The age distribution was as follows: 11.9% were aged between 60 and 70 years, 33.9% were aged between 70 and 80 years, and 54.2% were aged 80 years and older. The majority of the older adults from empty-nest households resided in rural areas (73%). Among them, 1,115 individuals (18.7%) exhibited signs of cognitive impairment. The characteristics of the participants are presented in Table 2.

**Table 2:** Characteristics of empty-nest older adult population (*n* = 5,961).

Demographic characteristics		Number of people	Percentage (%)	Mode
Gender	Male	3,079	51.7	Male
	Female	2,882	48.3	
Age	60 ~ 69	712	11.9	≥80
	70 ~ 79	2019	33.9	
	≥80	3,230	54.2	
Place of residence	City	1,608	27	Rural
	Rural	4,353	73	
Marital status	Have a spouse	3,667	61.5	Have a spouse
	No spouse	2,294	38.5	
Education status	Illiteracy	2,305	38.7	Non-illiterate
	Non-illiterate	3,656	61.3	
Cognitive impairment	Yes	1,115	18.7	No
	No	4,846	81.3	

### 3.2 Single factor analysis of cognitive impairment in empty-nest older adults

The results indicate that 4,456 respondents (75%) reside in bungalows, while 1,380 respondents (23%) reported that their houses are prone to leaks. Additionally, 2,360 respondents (40%) live within 200 meters of a main traffic road, and 1,816 respondents (30%) are located more than 3 kilometers from the nearest medical institution. Furthermore, 4,052 empty-nest older adults (68%) are exposed to chemical agents on a daily basis. Notably, 4,880 empty-nest older adults (82%) do not utilize piped natural gas, and 5,529 (93%) do not employ air purification devices. Finally, 4,735 empty-nest older adults (79.4%) primarily consume tap water.

The results of the single-factor analysis suggest that the cognitive functions of older adults residing in empty nests are influenced by various factors, including: (1) Housing conditions, such as the type of residence, housing type, presence of leaks, and proximity to major traffic routes; (2) Daily chemical exposure, involving contact with household chemicals; (3) Types of fuel used for daily living, including natural gas from pipelines, kerosene, coal, or firewood; (4) Indoor air quality, which encompasses kitchen ventilation, the presence of air purification devices, mold, smoking habits, and seasonal ventilation practices; and (5) Drinking water sources, referring to the type and category of drinking water. A Chi-square test indicated that 19 of these factors are statistically significant, implying a significant impact on the cognitive functions of older adults in empty nests and identifying potential influencing factors. For detailed information, please refer to Table 3.

**Table 3:** Analysis of univariate cognitive impairment in empty-nest older adults.

Variables	Total (*n* = 5,961)	No cognitive impairment (*n* = 4,846)	Cognitive impairment (*n* = 1,115)	Statistic	*p*
Place of residence, *n* (%)				χ^2^ = 80.845	<0.001
Rural	4,353 (0.73)	3,419 (0.71)	934 (0.84)		
City	1,608 (0.27)	1,427 (0.29)	181 (0.16)		
Housing type, *n* (%)				χ^2^ = 63.604	<0.001
Bungalow	4,456 (0.75)	3,519 (0.73)	937 (0.84)		
Apartment on 1 ~ 3 floors	275 (0.05)	239 (0.05)	36 (0.032)		
≥4 floors apartment without elevator	853 (0.14)	750 (0.15)	103 (0.092)		
≥4 floors apartment with elevator	345 (0.06)	310 (0.06)	35 (0.031)		
A mobile home	32 (0.0.01)	28 (0.01)	4 (0.004)		
Separate bedroom, *n* (%)				χ^2^ = 0.053	0.552
No	274 (0.05)	219 (0.05)	55 (0.05)		
Yes	5,687 (0.95)	4,627 (0.95)	1,060 (0.95)		
Leaking rain, *n* (%)				χ^2^ = 11.398	<0.001
No	4,581 (0.77)	3,767 (0.78)	814 (0.73)		
Yes	1,380 (0.23)	1,079 (0.22)	301 (0.27)		
Distance from the main road, *n* (%)				χ^2^ = 12.683	<0.001
≤200 meters	2,360 (0.4)	1971 (0.41)	389 (0.35)		
>200 meters	3,601 (0.6)	2,875 (0.59)	726 (0.65)		
Distance from medical institutions, *n* (%)				χ^2^ = 0.232	0.63
≤3 kilometers	4,145 (0.7)	3,363 (0.69)	782 (0.7)		
>3 kilometers	1816 (0.3)	1,483 (0.31)	333 (0.3)		
Exposure to daily chemical agents, *n* (%)				χ^2^ = 60.296	<0.001
No	1909 (0.32)	1,661 (0.34)	248 (0.22)		
Yes	4,052 (0.68)	3,185 (0.66)	867 (0.78)		
Use pipeline natural gas, *n* (%)				χ^2^ = 80.685	<0.001
No	4,880 (0.82)	3,863 (0.8)	1,017 (0.91)		
Yes	1,081 (0.18)	983 (0.2)	98 (0.09)		
Use kerosene, *n* (%)				χ^2^ = 37.642	<0.001
No	5,900 (0.99)	4,815 (0.99)	1,085 (0.97)		
Yes	61 (0.01)	31 (0.01)	30 (0.03)		
Use coal, *n* (%)				χ^2^ = 18.934	<0.001
No	5,761(0.97)	4,707(0.97)	1,054 (0.95)		
Yes	200(0.03)	139(0.03)	61 (0.05)		
Use firewood, *n* (%)				χ^2^ = 13.357	<0.001
No	4,121 (0.69)	3,401 (0.7)	720(0.65)		
Yes	1840 (0.31)	1,445 (0.3)	395(0.35)		
Kitchen ventilation, *n* (%)				χ^2^ = 75.966	<0.001
No	665 (0.11)	458 (0.09)	207 (0.19)		
Yes	5,296 (0.89)	4,388 (0.91)	908 (0.81)		
Use air purification device, *n* (%)				χ^2^ = 10.098	0.001
No	5,529 (0.93)	4,470 (0.92)	1,059 (0.95)		
Yes	432 (0.07)	376 (0.08)	56 (0.05)		
Moldy odor in the room, *n* (%)				χ^2^ = 44.568	<0.001
No	4,934 (0.83)	4,087 (0.84)	847 (0.76)		
Yes	1,027 (0.17)	759 (0.16)	268 (0.24)		
Smoking, *n* (%)				χ^2^ = 223.792	<0.001
No	3,629 (0.61)	3,170 (0.65)	459 (0.41)		
Yes	2,332 (0.39)	1,676 (0.35)	656 (0.59)		
Open windows for ventilation in spring, *n* (%)				χ^2^ = 90.138	<0.001
No	384 (0.06)	242 (0.05)	142 (0.13)		
Yes	5,577 (0.94)	4,604 (0.95)	973 (0.87)		
Open windows for ventilation in summer, *n* (%)				χ^2^ = 84.710	<0.001
No	199 (0.03)	112 (0.02)	87 (0.08)		
Yes	5,762 (0.97)	4,734 (0.98)	1,028 (0.92)		
Open windows for ventilation in autumn, *n* (%)				χ^2^ = 90.445	<0.001
No	340 (0.06)	210 (0.04)	130 (0.12)		
Yes	5,621 (0.94)	4,636 (0.96)	985 (0.88)		
Open windows for ventilation in winter, *n* (%)				χ^2^ = 92.954	<0.001
No	1,465 (0.25)	1,066 (0.22)	399 (0.36)		
Yes	4,496 (0.75)	3,780 (0.78)	716 (0.64)		
Category of drinking water, *n* (%)				χ^2^ = 6.979	0.008
Raw water	293 (0.55)	221 (0.05)	72 (0.06)		
Boiling water	5,668 (0.95)	4,625 (0.95)	1,043 (0.94)		
Type of drinking water, *n* (%)				χ^2^ = 24.712	<0.001
Well water	1,067 (0.179)	811 (0.167)	256 (0.23)		
River water or lake water	54 (0.009)	42 (0.009)	12 (0.011)		
Spring water	99 (0.017)	81 (0.017)	18 (0.016)		
Pond water	6 (0.001)	5 (0.001)	1 (0.001)		
Tap water	4,735 (0.794)	3,907 (0.806)	828 (0.743)		

χ^2^: Chi-square test.

### 3.3 Multifactorial analysis of cognitive impairment in empty-nest older adults

In this study, cognitive impairment was considered the dependent variable (0 = no, 1 = yes). Nineteen variables that demonstrated statistical significance in the univariate analysis were included in the binary logistic regression model for further analysis. A binary logistic regression analysis was conducted, revealing that factors such as residing in urban areas, utilizing natural gas from pipelines, ensuring kitchen ventilation, employing air purification devices, opening windows during winter, and consuming boiled water serve as protective factors for the cognitive function of empty-nest older adults. Conversely, exposure to daily chemical agents, the use of kerosene and coal, the presence of a musty odor in living spaces, and smoking were identified as risk factors for cognitive impairment among this demographic.

The probability of cognitive impairment among urban empty-nest older adults is relatively low (OR = 0.733, 95% CI: 0.577–0.930). Furthermore, the use of pipeline natural gas (OR = 0.600, 95% CI: 0.450–0.800), maintaining well-ventilated kitchens (OR = 0.683, 95% CI: 0.559–0.834), utilizing air purification devices (OR = 0.725, 95% CI: 0.534–0.983), opening windows for ventilation during winter (OR = 0.793, 95% CI: 0.668–0.941), and drinking boiled water (OR = 0.736, 95% CI: 0.549–0.986) are also associated with a lower probability of cognitive impairment.

Conversely, exposure to daily chemicals (OR = 1.595, 95% CI: 1.359–1.872), the use of kerosene (OR = 3.654, 95% CI: 2.117–6.308), the use of coal (OR = 1.523, 95% CI: 1.088–2.131), the presence of a musty smell in the room (OR = 1.352, 95% CI: 1.114–1.641), and smoking (OR = 2.606, 95% CI: 2.271–2.992) are all associated with a relatively high probability of cognitive impairment. Among these factors, the OR value for kerosene use is the highest, indicating a strong association with cognitive impairment. Refer to Table 4.

**Table 4:** Binary logistic regression analysis of factors affecting cognitive impairment in empty-nest older adults.

Variables	*β*	S.E	Waldχ^2^	*p*	OR (95%CI)
Place of residence (rural reference)					1.00 (reference)
City	−0.311	0.122	6.518	0.011	0.733 (0.577, 0.930)
Housing type (Bungalows Reference)			2.101	0.717	1.00 (reference)
Apartment on 1 ~ 3 floors	−0.173	0.192	0.816	0.366	0.841 (0.577, 1.225)
≥4 floors apartment without elevator	0.146	0.154	0.904	0.342	1.158 (0.856, 1.565)
≥4 floors apartment with elevator	0.053	0.215	0.061	0.806	1.054 (0.692, 1.607)
A mobile home	0.169	0.577	0.086	0.769	1.185 (0.382, 3.674)
Leaking rain (No reference)					1.00 (reference)
Yes	−0.047	0.093	0.258	0.612	0.954 (0.796, 1.144)
Distance from the main traffic road (≤200 meters reference)					1.00 (reference)
>200 meters	0.104	0.075	1.921	0.166	1.110 (0.958, 1.286)
Exposure to daily chemical agents (No reference)					1.00 (reference)
Yes	0.467	0.082	32.709	<0.001	1.595 (1.359, 1.872)
Use pipeline natural gas (No reference)					1.00 (reference)
Yes	−0.511	0.146	12.172	<0.001	0.600 (0.450, 0.800)
Use kerosene (No reference)					1.00 (reference)
Yes	1.296	0.279	21.638	<0.001	3.654 (2.117, 6.308)
Use coal (No reference)					1.00 (reference)
Yes	0.420	0.171	6.013	0.014	1.523 (1.088, 2.131)
Use firewood (No reference)					1.00 (reference)
Yes	−0.010	0.081	0.016	0.901	0.990 (0.844, 1.161)
Kitchen ventilation (No reference)					1.00 (reference)
Yes	−0.381	0.102	13.944	<0.001	0.683 (0.559, 0.834)
Use air purification device (No reference)					1.00 (reference)
Yes	−0.322	0.155	4.299	0.038	0.725 (0.534, 0.983)
moldy odor in the room (No reference)					1.00 (reference)
Yes	0.302	0.099	9.285	0.002	1.352 (1.114, 1.641)
Smoking (No reference)					1.00 (reference)
Yes	0.958	0.070	185.477	<0.001	2.606 (2.271, 2.992)
Open windows for ventilation in spring (No reference)					1.00 (reference)
Yes	−0.271	0.181	2.238	0.135	0.762 (0.534, 1.088)
Open windows for ventilation in summer (No reference)					1.00 (reference)
Yes	−0.359	0.248	2.092	0.148	0.698 (0.429, 1.136)
Open windows for ventilation in autumn (No reference)					1.00 (reference)
Yes	−0.209	0.206	1.030	0.310	0.811 (0.541, 1.215)
Open windows for ventilation in winter (No reference)					1.00 (reference)
Yes	−0.232	0.087	7.022	0.008	0.793 (0.668, 0.941)
Category of drinking water (Reference for raw water)					1.00 (reference)
Boiling water	−0.307	0.149	4.231	0.040	0.736 (0.549, 0.986)
Type of drinking water (Reference for well water)			1.753	0.781	1.00 (reference)
River water or lake water	0.098	0.357	0.076	0.783	1.103 (0.548, 2.222)
Spring water	−0.198	0.283	0.488	0.485	0.821 (0.471, 1.429)
Pond water	0.275	1.114	0.061	0.805	1.317 (0.148, 11.693)
Tap water	−0.097	0.089	1.189	0.276	0.907 (0.762, 1.081)

### 3.4 Establishment of Bayesian network model for cognitive impairment in empty-nest older adults

**Figure 2:**
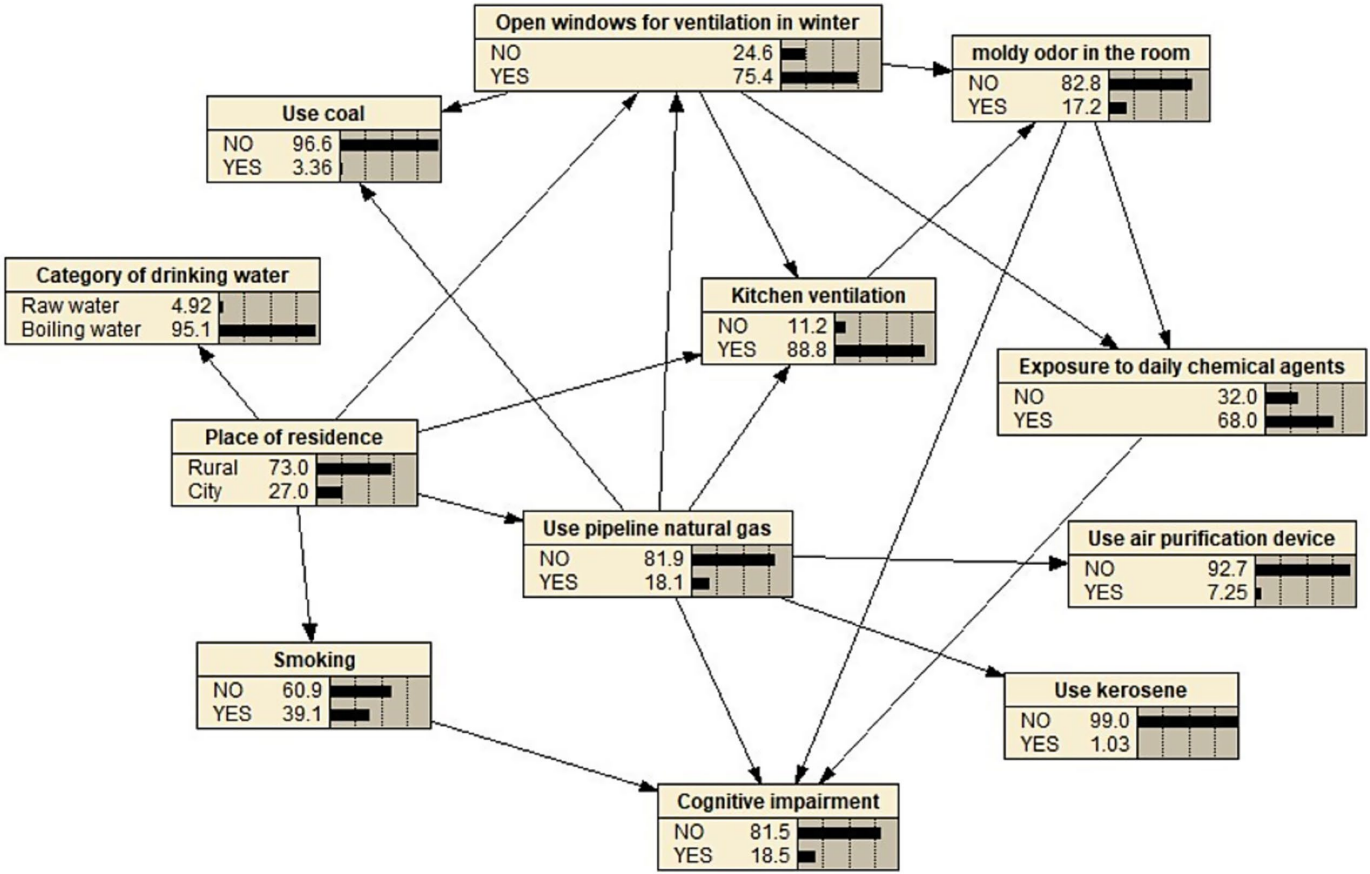
Topology of Bayesian network model for cognitive impairment in empty-nest older adults.

Based on the binary logistic regression analysis, eleven statistically significant variables were identified as network nodes. A Bayesian network model consisting of twelve nodes and twenty directed edges was constructed, and the conditional probabilities for each node were calculated. The cognitive impairment Bayesian network model demonstrates that factors related to the living environment are closely correlated with cognitive functions through multiple direct and indirect pathways, forming an interrelated and interactive network system. The results indicate that smoking, the use of natural gas from pipelines, the presence of a musty smell in the room, and exposure to daily chemical agents are directly associated with cognitive impairment. Conversely, factors such as living conditions, the type of drinking water, the use of coal, the opening of indoor windows during winter, kitchen ventilation, the use of air purification devices, and the use of kerosene are indirectly linked to cognitive impairment, suggesting that they represent indirect risk factors, as illustrated in Figure 2.

### 3.5 Reasoning of Bayesian network model for cognitive impairment in empty-nest older adults

**Figure 3:**
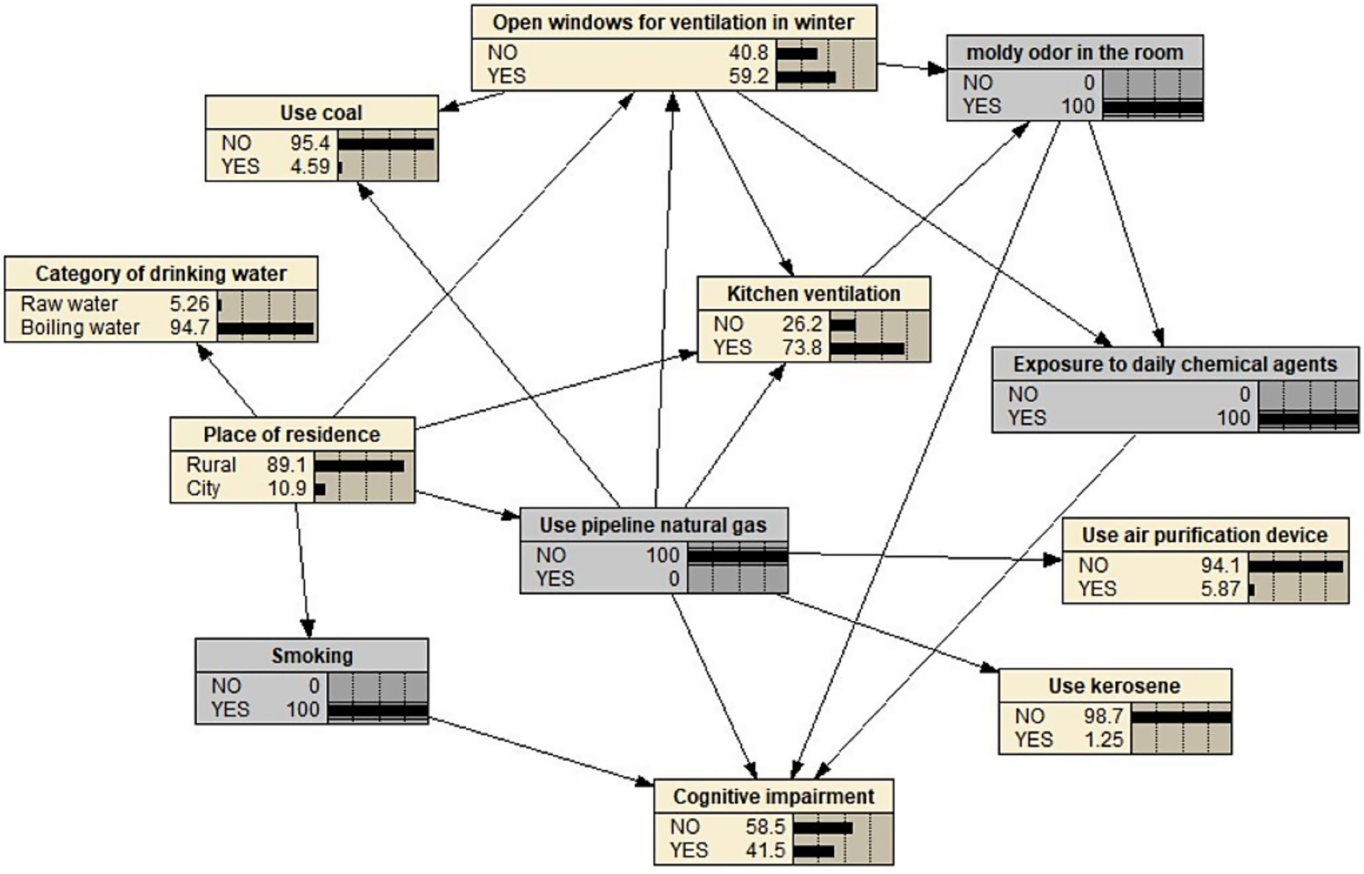
Topological diagram of cognitive impairment in empty-nest older adults who smoke, have moldy smell in the room, daily chemical contact, and do not use pipe natural gas.

The Bayesian network is utilized to predict risks, employing a Bayesian network probability prediction model constructed using Netica 5.18 software. Bayesian networks facilitate the inference of the probability of an unknown node based on the states of known nodes. By selecting the appropriate node to input information, one can deduce and confirm the risk of cognitive impairment among empty-nest older adults. The predicted probability of cognitive impairment varies with different combinations of exposure risk factors. Table 5 presents a conditional probability table with cognitive impairment as the central node, depicting the dependency relationships among smoking, the presence of a musty odor in the room, exposure to daily chemicals, and the use of pipeline natural gas, along with the conditional probabilities of cognitive impairment. The conditional probability table reveals that older adults living alone who smoke, have a musty odor in their rooms, are exposed to daily chemicals, and do not use pipeline natural gas have the highest risk of cognitive impairment (41.5%). Specifically, this is represented as P(cognitive impairment | smoking, musty odor in the room, exposure to daily chemicals, not using pipeline natural gas) = 0.415, as shown in Figure 3.

**Table 5:** Probability table for cognitive impairment in direct nodes using smoking, using pipe gas, musty smell, and chemical agents.

Whether to smoke	The musty smell in the room	Exposure to daily chemical agents	Use pipeline natural gas	Cognitive impairment	
				No (%)	Yes (%)
Yes	No	No	No	88.3	11.7
Yes	No	No	Yes	94.6	5.38
Yes	No	Yes	No	62.5	37.5
Yes	No	Yes	Yes	85.8	14.2
Yes	Yes	No	No	80.5	19.5
Yes	Yes	No	Yes	92.7	7.33
Yes	Yes	Yes	No	58.5	41.5
Yes	Yes	Yes	Yes	65.2	34.8
No	No	No	No	86	14
No	No	No	Yes	93.7	6.29
No	No	Yes	No	88.2	11.8
No	No	Yes	Yes	92.6	7.36
No	Yes	No	No	72	28
No	Yes	No	Yes	96.5	3.55
No	Yes	Yes	No	82.6	17.4
No	Yes	Yes	Yes	84.2	15.8

The conditional probability table clearly indicates a strong correlation between exposure to daily chemicals and the occurrence of cognitive impairment in empty-nest older adults. When an individual smokes, the room does not have a musty smell, there is no exposure to daily chemicals, and pipeline natural gas is not in use, the risk of cognitive impairment is 11.7%. This can be expressed as P(cognitive impairment | smoking, room without musty smell, no exposure to daily chemicals, not using pipeline natural gas) = 0.117. Under these same conditions, the incidence of cognitive impairment among empty-nest older adults rises from 11.7% (without exposure to daily chemicals) to 37.5% (with exposure to daily chemicals), represented as P(cognitive impairment | smoking, room without musty smell, exposure to daily chemicals, not using pipeline natural gas) = 0.375. The risk of cognitive impairment is significantly increased. Additionally, the use of pipeline natural gas correlates with a lower incidence of cognitive impairment. When the conditions of smoking, absence of a musty smell in the room, and lack of exposure to daily chemicals remain unchanged, the risk of cognitive impairment among empty-nest older adults decreases from 11.7 to 5.38% upon switching from not using to using pipeline natural gas.

## 4 Discussion

This study employed the Bayesian Network (BN) model to construct a complex probabilistic correlation network between environmental factors and cognitive impairment in empty-nest older adults. Given that this demographic often encounters unique challenges, such as limited social support and inadequate environmental maintenance capabilities (24), traditional regression models struggle to reveal the systematic interactions among multiple risk factors. The BN model utilized in this study, with its distinctive topological structure, clearly illustrates the multi-level and interdependent pathways among various influencing factors. This suggests that effective public health interventions for empty-nest older adults must adopt a systematic approach rather than merely focusing on the prevention and control of a single factor.

### 4.1 The correlation between core environmental factors and cognitive impairment

#### 4.1.1 Indoor moldy smell exposure: Bayesian network reveals its central role and neurotoxic mechanism in multiple environmental exposures

The Bayesian network model in this study demonstrates a direct correlation between moldy smell and cognitive impairment in empty-nest older adults. This node plays a critical role within the network, as it is linked both directly and indirectly to various environmental issues, such as insufficient ventilation and energy consumption, and also exhibits a statistical relationship with cognitive impairment. This topology suggests that a musty smell may serve as a convergence point for the failures of indoor environmental management systems. Furthermore, Bayesian networks imply its potential mediating role within complex networks of multiple exposures. Previous studies have indicated that mycotoxins and volatile organic compounds produced by mold metabolism may be associated with neuroinflammation (25, 26), which is often accompanied by neuronal damage and cognitive decline (27, 28). Conditional probability analysis indicated that, after controlling for other variables such as smoking, exposure to chemical agents, and the presence of pipeline natural gas, the risk of cognitive impairment among empty-nest older adults in environments with a moldy odor increased from 11.7 to 19.5%. Furthermore, the Bayesian network topology revealed that the moldy smell is associated with behavioral variables such as ‘winter window opening’ and ‘kitchen ventilation,’ as well as with ‘chemical agent use.’ It is hypothesized that exposure to moldy odors may coincide with certain coping behaviors among residents, such as the use of chemical fresheners or disinfectants, leading to a dual exposure scenario of ‘mold and chemical agents.’ Additionally, the data from this study indicated that 23% of empty-nest older adults experienced rain leakage in their homes, and 93% did not utilize air purification devices. This demographic shows limited awareness and ability to tackle environmental issues, leading to prolonged exposure to a high-humidity and mold-prone environment, which is difficult to rectify effectively. Consequently, based on the multiple correlation characteristics of moldy smell nodes revealed by the Bayesian network, the intervention strategy should shift from a singular focus on mold removal to a systemic response. This includes incorporating indoor environmental assessments into the health screening system for empty-nest older adults, promoting the use of portable humidity and mold detection equipment, and addressing housing conditions that may contribute to moldy odors. Specifically, efforts should concentrate on preventing leaks, improving ventilation, and providing dehumidification equipment to enhance social support for these individuals. Such measures may mitigate a range of health risks and offer new insights for the prevention of cognitive impairment. Additionally, it is essential to consider the unique characteristics of empty-nest households by enhancing peripheral social support, such as community resources, to compensate for the lack of support from children.

#### 4.1.2 Tobacco exposure: Bayesian network reveals independence and intervention potential of behavioral risk factors

In the Bayesian network model constructed for this study, there is a direct association exists between smoking behavior and cognitive impairment among empty-nest older adults individuals. Unlike traditional studies that merely report a superficial association between smoking and cognitive impairment, the Bayesian network model positions smoking as a direct risk node factor, independent of other environmental exposures (such as musty odors and the use of chemical agents). This suggests that the association between smoking and cognitive impairment may be both unique and significant. The conditional probability table indicates that, when controlling for other variables such as moldy smells, chemical agents, and lack of natural gas, the risk of cognitive impairment older adults who smoke in empty-nest situations increased from 15.8 to 34.8%. Notably, there is an edge connection between ‘place of residence’ and ‘smoking’ in the model, suggesting that geographical or socioeconomic factors may influence the distribution of smoking behavior. This further underscores the public health significance of targeting smoking as a key intervention for cognitive impairment. Existing literature indicates that harmful substances such as nicotine and carbon monoxide in tobacco may be related to pathophysiological processes, including cerebrovascular lesions, oxidative stress, and neuroinflammation, which in turn affect cognitive function (29–31). For empty-nest older adults, the persistence and difficulty in quitting smoking not only reflect their individual addiction characteristics but may also be influenced by social and psychological factors. These include insufficient social support, a lack of alternative activities, challenges in emotional regulation, and limited access to health information (32). Considering the independent and critical nature of smoking within the topological structure of the Bayesian Network (BN) model, it is proposed that interventions targeting this behavior may yield significant and independent health benefits. Empty-nest older adults typically encounter issues such as diminished social ties and weak support systems. Therefore, intervention strategies should prioritize enhancing social support and establishing a comprehensive tobacco prevention and control framework. In addition to implementing conventional measures such as policy advocacy, health education, and nicotine replacement therapy, there is a need to bolster supportive interventions at the community level. Respiratory physicians should be organized to conduct regular smoking cessation lectures within communities, and a “volunteer-older adult smokers living alone” pairing assistance mechanism could be established to provide ongoing behavioral support.

#### 4.1.3 Daily chemical agent exposure: Bayesian network reveals associations and potential mediating roles in environmental-behavior patterns

The Bayesian network structure in this study indicates that “daily chemical agent contact” serves as a crucial mediating node, directly linked to cognitive impairment and influenced by the two environmental factors: “winter window opening” and “moldy smell.” This topological relationship suggests that exposure to chemical agents may be a behavioral response to a poor indoor environment. From the changes observed in topological Figures 2, 3, it is evident that as the exposure levels to musty odors and inadequate winter ventilation increase, the likelihood of empty-nest older adults using chemical agents also rises, consequently leading to an upward trend in the risk of cognitive impairment within this population. Numerous studies have demonstrated that household chemicals, such as insecticides, mosquito repellents, and potent cleaners, may contain potential neurotoxins, including organophosphorus compounds. Prolonged exposure to these substances at low doses, especially in conditions of insufficient ventilation or limited personal protection, may be associated with neuronal damage and a decline in cognitive function. A French survey indicated that over 70% of women engage in house cleaning at least once a week (33), and the simultaneous use of multiple cleaning products is prevalent. Empty-nest older adults often undertake substantial housework independently, including deep cleaning and yard maintenance, which results in a higher frequency and dosage of exposure to potentially harmful substances (34). The Bayesian network model suggests an association pathway linking “musty smell,” “chemical agent use,” and “cognitive impairment.” It indicates that in response to adverse environmental conditions, the older adults may use air fresheners, disinfectants, or insecticides to address pest issues caused by musty odors or dampness, thereby increasing their exposure to neurotoxic substances. Consequently, intervention strategies should adopt a systematic approach: enhancing housing ventilation and moisture-proofing to mitigate musty odors at their source, while simultaneously strengthening community support. This includes promoting safer alternative products, such as physical pest control tools, and encouraging the use of protective equipment through community training, thereby supporting the cognitive health of empty-nest older adults from multiple dimensions.

#### 4.1.4 Pipeline natural gas use: analysis of systemic benefits of clean energy based on Bayesian network topology

The Bayesian network model presented in this study indicates that the ‘use of pipeline natural gas’ node is associated with a reduced risk of cognitive impairment, with this association being realized through multiple pathways. There exists a statistical dependency between this node and improvements in ‘kitchen ventilation’, the reduction of solid fuel usage, such as ‘coal’ and ‘kerosene’, and enhancements in indoor air quality. This intricate protective network is challenging to visually represent using traditional statistical models, thereby underscoring the methodological advantages of Bayesian Networks (BN) in identifying synergistic effects among multiple factors. As a clean energy source, pipeline natural gas can significantly decrease the emission of neurotoxic pollutants, including harmful indoor particulate matter (PM2.5/PM10), carbon monoxide, nitrogen oxides, and polycyclic aromatic hydrocarbons (35); In contrast to the combustion of solid fuels, it prevents the generation of high pollutant concentrations at the source. The generation of these pollutants (36, 37) has been shown to directly impair cognitive function (38, 39). The two Bayesian Network (BN) topological diagrams presented in the results section indicate that households utilizing pipeline natural gas exhibit a significantly lower probability of experiencing poor kitchen ventilation and reliance on solid fuels. When controlling for other variables, such as non-smoking, absence of musty odors, and non-use of chemicals, the risk of cognitive impairment among empty-nest older adults who utilized pipeline natural gas decreased from 14 to 6.9%. This finding suggests that clean energy may positively influence indoor air quality through multiple pathways. Vulnerable groups, such as empty-nest older adults, often encounter various challenges, including limited awareness of ventilation, prolonged exposure to pollutants, and inadequate social support (40, 41). The potential health benefits associated with the use of piped natural gas are particularly pronounced, as it may reduce the risk of falls caused by heavy objects, such as gas tanks or coal, and enhance cognitive functions by improving indoor air quality. Therefore, based on the network correlation structure revealed by the BN model, public health interventions should consider the promotion and subsidization of clean energy infrastructure as a crucial preventive strategy. It is recommended to reinforce targeted energy support for empty-nest older adults at the community level, integrate gas safety and indoor air quality assessments into health management practices, and establish household energy use records to mitigate health risks across multiple dimensions and enhance the potential benefits of clean energy in protecting cognitive health.

### 4.2 Indirect environmental factor association model based on Bayesian network: exploration of multi-path mediation and system intervention strategies

The Bayesian network model constructed in this study indicates that, in addition to the four direct correlation factors previously mentioned, “residential place” serves as a pivotal node. It is not only directly associated with factors such as drinking water type and smoking behavior, but also exhibits dependence on the energy structure represented by “pipeline gas use” and behavioral habits such as “winter window,” which may indirectly influence cognitive outcomes. This multi-level, multi-path correlation feature underscores the complexity of how residence impacts cognition. Previous studies have established that the risk of cognitive impairment among empty-nest older adults in rural areas is relatively high, attributed to factors including the allocation of primary medical and health resources (42), insufficient early screening for cognitive impairment (43), lack of parental companionship, and limited access to health services (44, 45). The Bayesian network topology diagram presented in this study further elucidates the correlation path from the perspective of “residence → drinking water type.” It also suggests that, based on prior research, drinking water should be regarded as a key intervention target, providing a foundation for the development of subsequent intervention measures.

Traditional solid fuels, such as coal and kerosene, are significant sources of exposure in the living environment, and their usage is closely linked to regional differences in energy infrastructure. For empty-nest older adults, who often have weak social support and limited economic resources, there is a greater likelihood of prolonged reliance on these inexpensive fuels. This results in higher exposure to indoor air pollution, which aligns with previous research findings (46–48). Furthermore, the model indicates that ‘winter window opening’ serves as a behavioral node with complex correlation patterns. This behavior is associated with improved kitchen ventilation, reduced musty odors, and decreased accumulation of chemical agents, while also being related to fluctuations in indoor temperature. This suggests that ventilation behavior may involve a trade-off between comfort and health benefits for empty-nest older adults. individuals. Air purification devices serve a protective and regulatory function. Research indicates that maintaining a conducive ventilation and temperature environment can enhance cognitive stability (16). Given that empty-nest older adults spend approximately 85% of their daily lives indoors (49), the impact of indoor air quality on cognitive health is particularly crucial. Therefore, based on the correlation network revealed by the BN model, intervention measures should be systematized: on one hand, promoting the transition to clean energy to reduce pollution exposure at the source; on the other hand, communities should install affordable exhaust systems in older homes and incorporate ventilation frequency assessments into home safety visits, thereby safeguarding the cognitive function of empty-nest older adults through overall improvements in indoor air quality.

### 4.3 Identification of high-risk populations based on Bayesian network: multi-risk factor combination pattern and precise prevention implications

An important methodological advantage of the Bayesian network model is its ability to identify and quantify synergistic risk patterns within multi-factor combinations. Its unique topology provides a solid foundation for accurately pinpointing multi-factorial synergistic risks. First, the model identifies the riskiest combination of factors based on this topology: the conditional probability table indicates that when an individual exhibits various characteristics such as “smoking,” “moldy smell in the room,” “exposure to daily chemical agents,” and “unused pipeline natural gas,” the risk of cognitive impairment reaches 41.5%. This risk is significantly higher than that associated with any single factor or simple two-factor combination, thus providing a clear target for accurately identifying high-risk groups. More importantly, the Bayesian network model performs conditional probability analysis based on the relationships defined by its topological structure, revealing non-additive collaborative dependencies among factors. For instance, if “exposure to daily chemical agents” can be mitigated by eliminating exposure, the risk of cognitive impairment can be significantly reduced from 41.5 to 19.5%. A similar decrease in risk was observed by addressing the “musty smell in the room” alone or by promoting the use of “pipeline gas.” This demonstrates that the effect of this high-risk combination is not merely a simple aggregation of multiple factors; rather, it arises from the complex conditional dependencies inherent in the topology. Changes in specific factors can significantly alter the final probability of cognitive impairment, given the presence of other contributing factors. This finding presents a novel perspective for developing intervention strategies, emphasizing that even if not all risk factors can be immediately modified (such as long-term smoking), prioritizing controllable environmental factors (like eliminating musty odors from homes and promoting clean energy) can sever critical links in the risk chain. This approach may yield substantial benefits for the cognitive function of older adults living alone. The intervention logic, which focuses on ‘prioritizing the resolution of variable factors,’ is not attainable through traditional regression analysis. Consequently, the Bayesian Network (BN) model used in this study not only facilitates the precise identification of high-risk populations but also provides a scientific foundation for crafting personalized intervention strategies based on the prioritization of risk portfolios. This analysis leverages topological structure and conditional dependency relationships, showcasing the value of Bayesian networks in transcending conventional multi-factor analysis within complex systems such as living environments and health. They offer dynamic and systematic insights that traditional multi-factor analysis cannot provide, aiding public health professionals in accurately defining high-risk group profiles and achieving ‘precise prevention.’

### 4.4 Limitations

This study acknowledges several limitations. First, it lacks empirical research on the cognitive disorders of empty-nest older adults based on a Bayesian network model of living environments. Second, the data utilized in this study are self-reported by the participants, which introduces potential recall and reporting biases. Finally, this study employs cross-sectional data, and the causal pathways identified require further verification through prospective studies.

## 5 Conclusion

The incidence of cognitive impairment among the empty-nest older adults is notably high. Contributing factors include living in rural areas, exposure to daily chemical agents, the absence of piped natural gas, reliance on kerosene and coal for heating, inadequate kitchen ventilation, lack of air purification devices, the presence of moldy odors in living spaces, smoking, insufficient window ventilation during winter, and consumption of untreated water. This study employs Bayesian network modeling to thoroughly investigate the intricate mechanisms by which environmental factors influence cognitive impairment among the empty-nest older adults. Unlike traditional logistic regression models, Bayesian networks integrate the topological relationships between probability calculations and visualizations, thereby facilitating a clear analysis of the complex probabilistic dependencies and interaction networks among various factors and their association with cognitive impairment. This approach enables precise identification of specific environmental risk factors, protective factors, and their independent effect intensities. Consequently, it aids medical professionals in implementing targeted intervention measures for high-risk groups promptly, thereby mitigating the onset and progression of cognitive impairment. Ultimately, this research aims to provide a scientific basis and data support for the development of more accurate and effective cognitive health interventions tailored to specific living environments, as well as for the establishment of ‘cognitively friendly’ communities for the older adults.

## Data Availability

Publicly available datasets were analyzed in this study. This data can be found here: this study analyzed publicly available datasets. The data supporting the results can be accessed at the following website: https://opendata.pku.edu.cn/dataverse/CHADS.
